# Roles of immune microenvironment in the female reproductive maintenance and regulation: novel insights into the crosstalk of immune cells

**DOI:** 10.3389/fimmu.2023.1109122

**Published:** 2023-12-28

**Authors:** Mengyuan Dai, Ying Xu, Guidong Gong, Yaoyao Zhang

**Affiliations:** ^1^ Department of Obstetrics and Gynecology, Key Laboratory of Birth Defects and Related of Women and Children of Ministry of Education, West China Second University Hospital, Sichuan University, Frontier Medical Center, Tianfu Jincheng Laboratory, Chengdu, Sichuan, China; ^2^ National Engineering Laboratory for Clean Technology of Leather Manufacture, Sichuan University, Chengdu, China

**Keywords:** female fertility, immune microenvironment, single-cell sequencing, reproductive process, maternal-fetal interface

## Abstract

Female fertility decline is an accumulative consequence caused by complex factors, among them, the disruption of the immune profile in female reproduction stands out as a crucial contributor. Presently, the effects of immune microenvironment (IME) on the female reproductive process have attracted increasing attentions for their dynamic but precisive roles. Immunocytes including macrophages, dendritic cells, T cells, B cells and neutrophils, with diverse subpopulations as well as high plasticity functioned dynamically in the process of female reproduction through indirect intercellular communication via specific cytokine release transduced by molecular signal networks or direct cell-cell contact to maintain the stability of the reproductive process have been unveiled. The immune profile of female reproduction in each stage has also been meticulously unveiled. Especially, the application of single-cell sequencing (scRNA-seq) technology in this process reveals the distribution map of immune cells, which gives a novel insight for the homeostasis of IME and provides a research direction for better exploring the role of immune cells in female reproduction. Here, we provide an all-encompassing overview of the latest advancements in immune modulation within the context of the female reproductive process. Our approach involves structuring our summary in accordance with the physiological sequence encompassing gonadogenesis, folliculogenesis within the ovaries, ovulation through the fallopian tubes, and the subsequent stages of embryo implantation and development within the uterus. Our overarching objective is to construct a comprehensive portrayal of the immune microenvironment (IME), thereby accentuating the pivotal role played by immune cells in governing the intricate female reproductive journey. Additionally, we emphasize the pressing need for heightened attention directed towards strategies that focus on immune interventions within the female reproductive process, with the ultimate aim of enhancing female fertility.

## 1 Introduction

The fertility rate decline has emerged as a global public health concern, carrying the potential to disrupt population dynamics and exert adverse impacts on worldwide economic progress (1). Data furnished by Organization for Economic Co-operation and Development (OECD) have shown a dramatic decline in fertility rates during the past few decades among more than 50 countries around the world, including China. Chinese fertility rates calculated by children/women remained below 2.0 for the past 10 years, which indicates negative population growth. Fertility decline in females is regarded as the most critical reason leading to the reduction in the fertility rate. Despite the physical factors of postponed childbearing age (2) and excessive obesity of women (3), abnormal immunomodulation in the female reproductive process was considered the central cause of female infertility (4).

The IME can be envisioned as the native habitat where immune cells reside, underscoring the significance of intercellular communication and comprehensive coordination. Owing to the distinctive physiological traits of the female reproductive system, its immune system demonstrates complexity and variability, a subject that has captivated the attention of numerous researchers. Furthermore, the equilibrium of IME within female reproduction has been highlighted for its pivotal contribution to the entirety of the female reproductive journey. Despite the presence of several reviews encapsulating the fluctuating dynamics of the immune system during various female physiological or pathological processes (5–8), there is still a lack of systematic summary to give a comprehensive description of the dynamic alteration for the whole female reproductive process. Moreover, aided by advanced high-throughput technologies, particularly the integration of scRNA-seq, a multitude of immune sub-populations and their diverse functions within the orchestration of the female reproductive process have come to light (9). we present a comprehensive overview of the latest advancements in understanding immune cell involvement throughout the female reproductive journey. Our overarching objective is to construct a holistic regulatory network that unveils the role of IME in female reproduction, while shedding light on the intricate web of communications established between immune cells and the localized microenvironment. This review aspires to provide insights that will contribute to the enhancement of research and the development of immune modulation-based therapies, tailored to amplify female fertility.

## 2 Roles of IME involved in gonadogenesis, folliculogenesis and ovulation

### 2.1 Mφs’ involvement in the process of gonadogenesis

Within the female context, oocytes trace their origins back to oogonia, derived from primordial germ cells through a process of differentiation (10). Gonad development is a highly regulated process that coordinates cell size and morphogenesis to produce the sex-specific organ structure needed for reproduction. Sex differentiation is obvious in the whole organ, including the interstitial compartment containing immune cells and the vascular system (11).

The nascent immune cells in proximity to the gonad of the neonatal fetus comprise primitive macrophages (Mφs), pivotal entities that wield significant influence over various facets of gonadogenesis and fertility. Their roles encompass diverse responsibilities, such as orchestrating the establishment and sustenance of sexual gonadal vasculature across different phases of life (12). During fetal development and up to birth, additional immune cell types, including monocytes and a limited population of eosinophils, can be identified within the gonadal milieu. Nonetheless, the precise contributions of these cells to ovarian organogenesis and functionality remain largely enigmatic (13).

**Figure 1:**
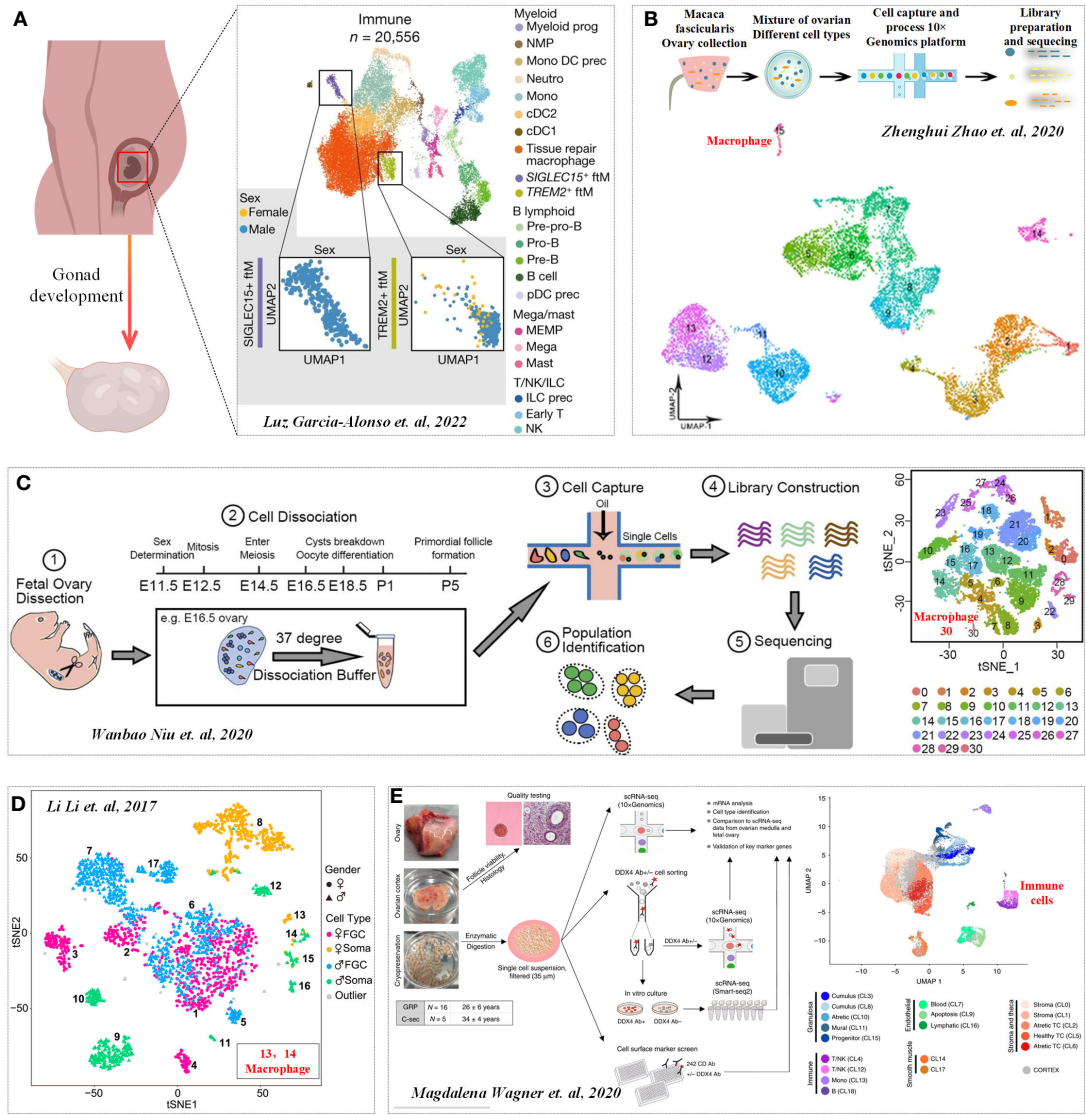
Immune profile in Gonadogenesis. **(A)** UMAP of immune cell states (color) in the human scRNA-seq data. Cited from Luz Garcia-Alonso et al, 2022 (15). b-d. Distribution of Mφs in monkeys **(B)**, mice **(C)** and humans fetal ovaries **(D)**. Cited from (16–18). **(E)** Transcription profiles of six main cell types in ovarian tissue. Cited from Magdalena Wagner et al, 2020 (19).

During the development of mouse embryos, the ovarian Mφs at E14.5 mainly included dominant CD11b^Int^F4/80^Hi^ cells, while the number of CD11b^Hi^F4/80^Int^ cells in E16.5 ovaries increased significantly, indicating that the Mφs population changed significantly (14). In addition, CD11b^Hi^F4/80^Int^ Mφs expressed monocyte markers, such as LY6C and CCR2 (M2 marker), but MHCII (M1 marker) was not expressed in the two Mφs populations, which indicated that fetal ovarian Mφs showed M2-type Mφs (14). In addition to immunophenotyping, scRNA-seq has been widely used to clarify the dynamic process of tissue development. The latest scRNA-seq study on gonad development also illustrates the crucial regulatory signals for gonad formation and the dynamic process of gonadal development. While the role of immune cells is not elaborated upon in the process of female gonad formation and development, a discovery was made during the process of male gonad formation. It was found that macrophages double-positive for SIGLEC15+ and TREM2+ can determine gonad formation by regulating somatic cells (**Figure 1A**) (15). Previous studies have also revealed the distribution of Mφs as well as other immune cells during the development of different species (including mice, monkeys and humans) of fetal ovaries (**Figures 1B–D**) (16–18). However, the effect of these Mφs on fetal ovaries (including angiogenesis and development) has not yet been revealed, which may be an important research direction in the future.

### 2.2 The role of Mφs in the formation of follicles

The ovary serves as the focal point of folliculogenesis, undertaking the dual responsibilities of oocyte production and differentiation, as well as the release of sex hormones. The integration of scRNA-seq technology has ushered in a new era in the study of follicular formation and development, transitioning from an emphasis on singular cellular events to a simultaneous exploration of the microenvironment within the entire follicle (20). Through a scRNA-seq investigation, the transcriptional profiles of six primary cell types within ovarian tissue were unveiled: oocytes, granulosa cells, immune cells, endothelial cells, perivascular cells, and stromal cells (**Figure 1E**) (19). Furthermore, granulosa cells, a key player across various developmental stages of ovarian follicles, were comprehensively studied through another single-cell sequencing study (21). Regulatory entities like immune cells also hold sway in governing the microenvironment (22). It’s notable that any disruption in the balance of the immune microenvironment (IME) can trigger ovarian dysfunctions (8, 23).

Mφs are the dominant immunocytes in ovaries, with the characteristics of high heterogeneity and plasticity in their functions when responding to diverse stimulations (24). In general, according to the exposure signals, Mφs can be polarized into the proinflammatory M1 type and anti-inflammatory M2 type. M1 Mφs mainly function in eliminating intracellular pathogens, whereas M2 Mφs participate in tissue remodeling and repair, as well as the resolution of inflammation (7, 25). Mφs are found to regulate folliculogenesis. Researchers constructed M1-like CD11c DTR mice (CD11c depletion mice) and M2-like CD206 DTR mice (CD206 depletion mice) to investigate the role of M1-like Mφs and M2-like Mφs in folliculogenesis. Compared with WT mice, folliculogenesis was impaired. In CD206 DTR mice, folliculogenesis was normal, and the ovulation number, fertilization rate, and implantation rate were similar to those in WT mice, indicating the necessity of M1 populations in folliculogenesis (26). Nevertheless, Luba et al. observed no significant change in ovarian follicle numbers, follicle atresia, or apoptosis within 5-21 days post-depletion of CX3CR1 in the Wistar rat model that allows a conditional depletion of circulating monocytes, despite an effective depletion of ovarian monocytes and monocyte-derived Mφs (27).

During a menstrual cycle, despite there are several follicles at different developmental stages in the ovary, there is usually only one; in rare cases, two follicles can develop into larger dominant follicles and participate in the subsequent ovulation process. The fate of a primordial follicle hinges upon a decisive choice: to be a dominant follicle ovulated or atresia follicle phagocytosed by Mφs. Primordial follicle development unfolds in two distinct waves, each characterized by varying rates of progression. It is likely that the fast-growing first wave of follicles facilitates the establishment of the hypothalamic–pituitary–ovarian axis and thereby plays a key role in the onset of puberty and the initiation of reproductive life, while the second wave of relatively slow-growing adult primordial follicles contribute to continuous ovulation throughout the middle and late stages of reproductive life (28). Prior research indicates that the initial stages of follicle growth appear to be independent of Mφ influences because direct interactions between Mφs and primordial follicles have not been observed. However, an increase in the number and localization change to the theca cell layer formed at the secondary follicle stage of ovarian Mφs in healthy follicles was observed (29), which suggests that Mφs indeed participate in the process of follicle growth and development in a paracrine manner. Liu et al. highlighted the importance of Mφs in primordial follicular selection and discovered the potential functions of immune response genes and the NF-κB pathway on primordial follicular selective activation in bovine ovary (30). Interestingly, in accordance with the results found by Yosuke Ono (26), Xiao et al. observed the stimulatory effects of M1-like Mφs and surprising inhibitory effects of M2-like Mφs, while no obvious effect of M0-like Mφs on primordial follicles was observed by coculture of both newborn ovaries with M0, M1 and M2-like Mφs and exocellular vehicles derived from them, suggesting that M1-like Mφs were necessary and promotive for primordial folliculogenesis and subsequent selective activation. Mechanistically, the levels of p-AKT and p-RPS6 in M1-treated ovaries were elevated, while both phosphorylated proteins were markedly decreased in the M2 group. In addition, they found that two specific miRNAs, miR-107 from M1-EVs and miR-99a-5p from M2-EVs, participated in modulating this signaling pathway and follicular selective activation in an opposing manner by targeting PTEN and mTOR, respectively (**Table 1**) (7). Furthermore, infiltration of Mφs in the ovary can also be modulated by several endogenous or exogenous factors and leads to disorders of ovarian function (31, 32).

**Table 1:** Types and functions of immune cells involved in folliculogenesis.

Cell type	Model or mechanism	Functions	References
**M1 like Mφs**	CD11c depletion mice	Positive relations with primordial follicle formation	(26)
	Up-regulation of p-AKT, PI3K, p-RPS6, mTOR	Promoting primordial follicle selective activation	(7)
**M2 like Mφs**	CD206 depletion mice	Not necessary for primordial follicle formation	(26)
	Down-regulation of p-AKT, PI3K, p-RPS6, mTOR	Inhibiting primordial follicle selective activation	(7)
**Monocyte**	CX3CR1 depletion Wistar rat model	Not necessary for primordial follicle formation	(27)
**Total Mφs**	NF-κB	Necessary for primordial follicle selective activation	(30)

### 2.3 Activation of immune cell infiltration in preovulatory follicles

The main mission for preovulation follicles is to prepare for ovulation; thus, a series of molecular events to promote oocyte meiotic resumption, remodel the follicle structure, and prepare to support luteal function are needed in this stage (33, 34). As a part of ovarian tissue remodelling and repair during ovulation follicular rupture and oocyte release, it is a physiological inflammatory reaction involving sex hormones in cooperation with various proteases and cytokines (35, 36). Follicular fluid (FF) contains a variety of cytokines and immune cells, including IL6, IL12, sHLA-G, Mφs, NK cells and dendritic cells (DCs) (37). Therefore, changes in IME balance are closely related to changes in follicular development, oocyte maturation, oocyte quality and ovulation.

**Figure 2:**
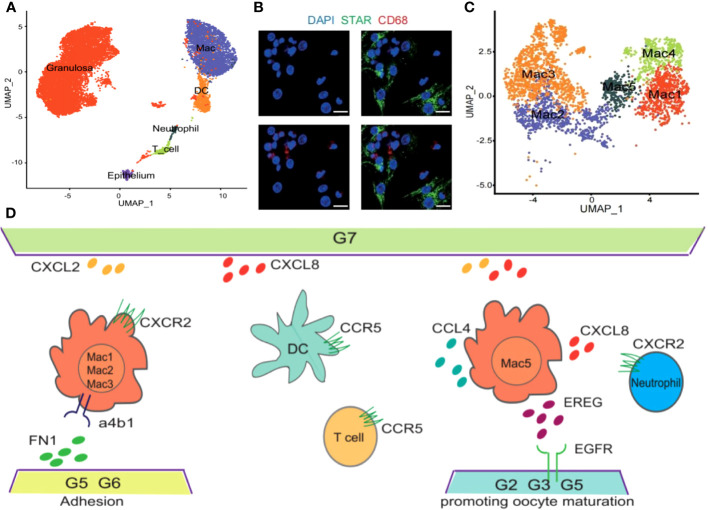
Immune profile in preovulatory follicles. Cited from Huihua Wu et al, 2022 (38). **(A)** UMAP showing immune cell types in preovulatory follicles. **(B)** Immunofluorescence showing the existence of GCs (STAR, green) and macrophages (CD68, red) in preovulatory follicles. DAPI (blue)-labelled cell nucleus. **(C)** Five different clusters (M1–M5) identified in the macrophage population, shown in a UMAP. **(D)** Scheme of the main ligand and receptors’ interaction of GCs and immune cells.

Owing to the challenges associated with obtaining preovulatory follicles during a natural menstrual cycle, research centered on this specific phase remains relatively constrained. Evidence from scRNA-seq conducted by Wu et al. has demonstrated the congregation of various immune cell types including Mφs, DCs, T cells, as well as neutrophils within preovulatory follicles and actively participate in the preparation for follicular ovulation (**Figure 2A**). Moreover, through immunofluorescence analysis, it was revealed that Mφs (positive for CD68) infiltrate the vicinity of granular cells (positive for STAR), underscoring their regulatory role in the subsequent ovulation process (**Figure 2B**). In addition, a total of five clusters of Mφs (Mφ1, Mφ2, Mφ3, Mφ4, Mφ5) within preovulatory follicles were observed and verified by immunofluorescence (**Figure 2C**), which have a variety of functions during ovulation (38). In detail, M2-like Mφ1 has anti-inflammatory effects, inflammation-responsive Mφ2, undifferentiated and immature Mφ3, Mφ4 with a high secretion of exosomes to degrade and remodel the extracellular matrix (ECM), and Mφ5 associated with granulocytes and cell chemotaxis. Collectively, the different populations of Mφs in preovulatory follicles exhibit obvious heterogeneity in their expression profiles as well as functions (38). It is interesting that the communications between Mφs and granulosa cells were uncovered. They found that there were strong interactions between granulosa cells and Mφs involved in cytokine–cytokine receptor interactions, cell adhesion molecules, the chemokine signaling pathway and the EGFR tyrosine kinase pathway (38). All these interactions collectively stimulate Mφs chemotaxis, leading them to adhere to and inhabit the preovulatory follicles, which have been categorized into nine distinct groups (G1-G9) based on their varied roles in biological processes. Additionally, Mφs also closely communicate with DCs via the interaction of CCL4 and its receptor CCR5, with T cells via the interaction of CCL4L2 and PGRMC2, CCL4 and CCR5, with neutrophils via the interaction of CXCL2 and CXCR2, CXCL8 and CXCR2/CXCR1, CXCL3 and CXCR2, and CCL3 and CCR1, indicating a role for Mφs in DC, T-cell and neutrophil recruitment (**Figure 2D**) (**Table 2**) (38). Inevitably, these alterations in cellular signals were also preparations for subsequent ovulation. However, due to the limitations of single-cell sequencing, which primarily reveals presence, further experimental validation is required to establish causality. This point is also supported by the table summarizing the content of the immune-related single-cell sequencing studies we referred to (**Table 3**). As a result, the temporal expression of these cytokines and the competition among immune cells with the same receptors expressing them are worthy of investigation, and the functions excluded by these infiltrated immune cells is with necessity and meanings to be clarified. Studies has shown that immune cells can participate in follicle growth, oocyte maturation, ovulation and luteinization by secreting cytokines and chemokines (39, 40). Moreover, they can also facilitate tissue repair after ovulation via phagocytosis (41, 42). However, the underlying mechanisms are also needed to be elucidated.

**Table 2:** Cytokine-receptor interactions.

Cytokines from Mφ	Receptors	Cell expression	Functions
CCL4	CCR5	DCs	DCs recruitment
CCL4	CCR5	T cells	T cells recruitment
CCL3	CCR1	Neutrophils	Neutrophils recruitment
CXCL2	CXCR2		
CXCL3	CXCR2		
CXCL8	CXCR1/2		

**Table 3:** Summary of immune related scRNA-seq articles.

No.	Sample	Species	Immune cell	Marker	Functions	Ref
1	Fetal ovary	Monkey	Mφs	TYROBP	/	(16)
2	Gonadal	Human	Mφs	CD68 SIGLEC15	Sex determination	(15)
				CD68 TREM2		
				CD68 F13A1	Tissue-repairing	
3	Ovarian cortex	Human	T cells	CD2 CD3G CD8A	/	(20)
			Antigen-presenting dells	CD14 HLA-DRA		
				B2M HLA-DQB1		
4	Preovulatory Follicular	Human	Mφs	CD68 PTPRC	Degradation and remodeling of ECM/Anti-inflammation /Inflammatory response	(38)
			DCs	CD1C PTPRC	/	
			Neutrophils	CXCR2 PTPRC		
			T cells	CD3D CD3E PTPRC		

### 2.4 Immune activation during ovulation

**Figure 3:**
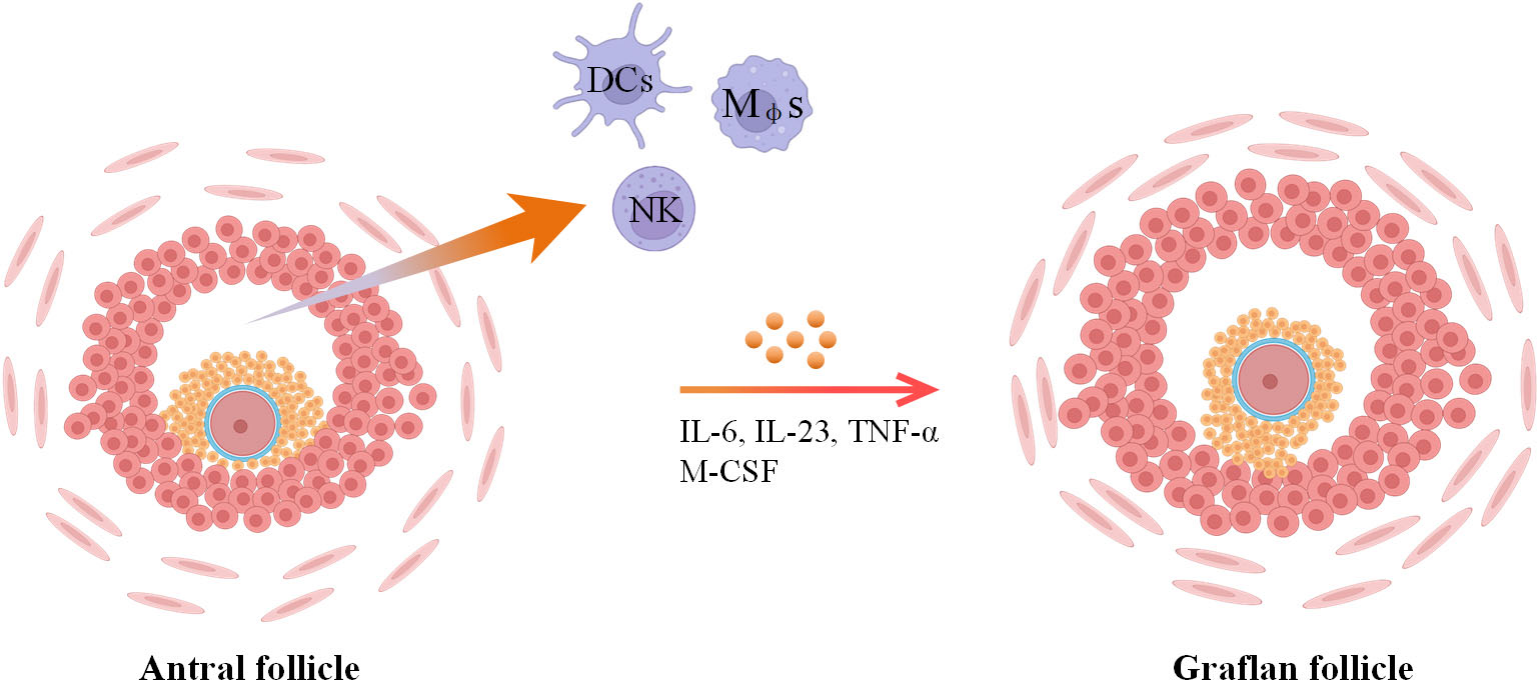
Role of immune cells during ovulation. Summarized from (22) and (47).

Ovulation stands as a pivotal milestone for the attainment of successful pregnancy and is underpinned by intricate cellular and molecular networks. Research has unveiled that both resident and migrating immune cells play a contributory role in facilitating the achievement of successful ovulation. In more detailed investigations, numerous studies have demonstrated that ovulation can be induced in perfused rabbit and rat ovaries in *in vitro* models, indicating that supplements of migrated immune cells are not required for ovulation (43–45). However, ovulation efficiency is decreased in this model. Another finding declared that the ovulation rate can be increased by leukocyte supplementation in *in vitro* perfused rat ovaries (46). Consequently, it could be posited that immune cells residing within the ovaries hold the capacity to initiate ovulation, yet to attain complete ovulatory potential, the influx of additional immune cells becomes requisite (6). During ovulation, the surge of luteinizing hormone (LH) can first act on granulosa cells and theca cells to produce inflammatory mediators and then activate immune cells (6). Preovulatory DCs are an important component of immune cells, and the maturity of DCs is positively correlated with the ovarian response to gonadotropin. During ovulation, these immune cells (distributed in follicular fluid) and granulosa cells can produce a large number of inflammatory factors (IL-6, IL-23 and TNF-α) and interact with each other to regulate ovarian function (**Figure 3**) (6, 47).

Degradation of ECM at the follicular apex is a key event for ovulation to be successful in enabling the release of the oocyte. Coincidentally, neutrophils are believed to actively participate in ECM remodeling by secreting proteases such as MMP-9, MPO and NE (48), supported by the observation that the ovulation rate was reduced in neutrophil-depleted rats (49, 50). Therefore, the rearrangements of cytokines and immune cells infiltrating the follicles are the foundation for this structural change to achieve ovulation. Notably, the cytokines expressed by Mφs function to remodel the ECM by releasing cathepsin family members CTSD, CTSB, and CTSL (51), which are essential for follicle rupture. Among them, CTSD is capable of initiating a proteolytic cascade and remodeling the ECM (52); moreover, the local accumulation of CTSB and CTSL in Mφs is associated with the degradation of tissues during inflammatory responses (53).

Infiltrations of immune cells are necessary for ovulation and are modulated by multiple cells resident in preovulation follicles by releasing chemokines (47). For example, TECK is secreted by ovarian resident cells surrounding mature follicles and localizes to the theca layers of follicles to recruit monocytes (54), which are responsible for removing damaged cells and tissues via phagocytosis (41, 42). Mφs are the dominant cells resident in follicles and the immune population chemotaxis into preovulation follicles (**Figure 3**) (22), which play important roles in ovulation. Mice with lower monocyte/macrophage counts by mutation in the M-CSF gene have reduced ovulation rates (55). Additionally, the administration of clodronate liposomes into the ovarian bursa of the mouse (56) or M-CSF neutralizing antibodies (57) into the bursa of the primed rats to deplete the ovary from Mφs can lead to a decrease in ovulation rates. Furthermore, Mφs possess the capability to release MCP-1 within the perifollicular stroma in the late ovulatory phase to Mφs induce macrophage chemotaxis and contribute to attracting mast cells and T lymphocytes or merely activating tissue-bound Mφs to facilitate ovulation (42). Among the attracted populations, mast cells have been identified to secrete IL-8, a cytokine that is thought to foster follicle growth and bolster ovulation rates *in vitro* (58) and further to induce neutrophil accumulation and activation in ovulation (**Table 4**) (59). Moreover, granulosa cells in preovulatory follicles are also contributors to ovulation by expressing Pgr to attenuate excessive ovulatory inflammation by diminishing Ptgs2 expression (60), which promotes inflammation by producing proinflammatory prostaglandins (61). Abnormal NK cells in FF will also affect the development of follicles (**Figure 3**), but its specific molecular mechanism has not been revealed. In addition, CXCL12 secreted by granulosa cells is another chemokine that helps T lymphocyte chemotaxis to preovulation follicles to regulate ovulation and conversely reduces the apoptosis of granulosa cells with the help of T lymphocytes (62). Here, we consolidate the diverse intercellular communications facilitated by cytokines and pathways, aiming to provide a comprehensive elucidation of the intricate alterations occurring within follicles during the process of ovulation.

**Table 4:** Types and functions of immune cells involved in ovulation.

Cell type	Cytokines	Functions	References
**Dendritic cells**	IL-6 IL-23 TNF-α	Ovarian response to gonadotropin	(6, 47)
**Neutrophils**	MMP-9 MPO NE	Enhancing ECM remodeling	(48–50)
**Macrophages**	CTSD CTSB CTSL	Enhancing ECM remodeling	(51–53)
	MCP-1	Recruiting mast cells T cells	(42)
**Mast cells**	IL-8	Enhancing follicle Growth and ovulation Neutrophils accumulation	(58, 59)

## 3 Roles of IME in endometrial receptivity regulation

**Figure 4:**
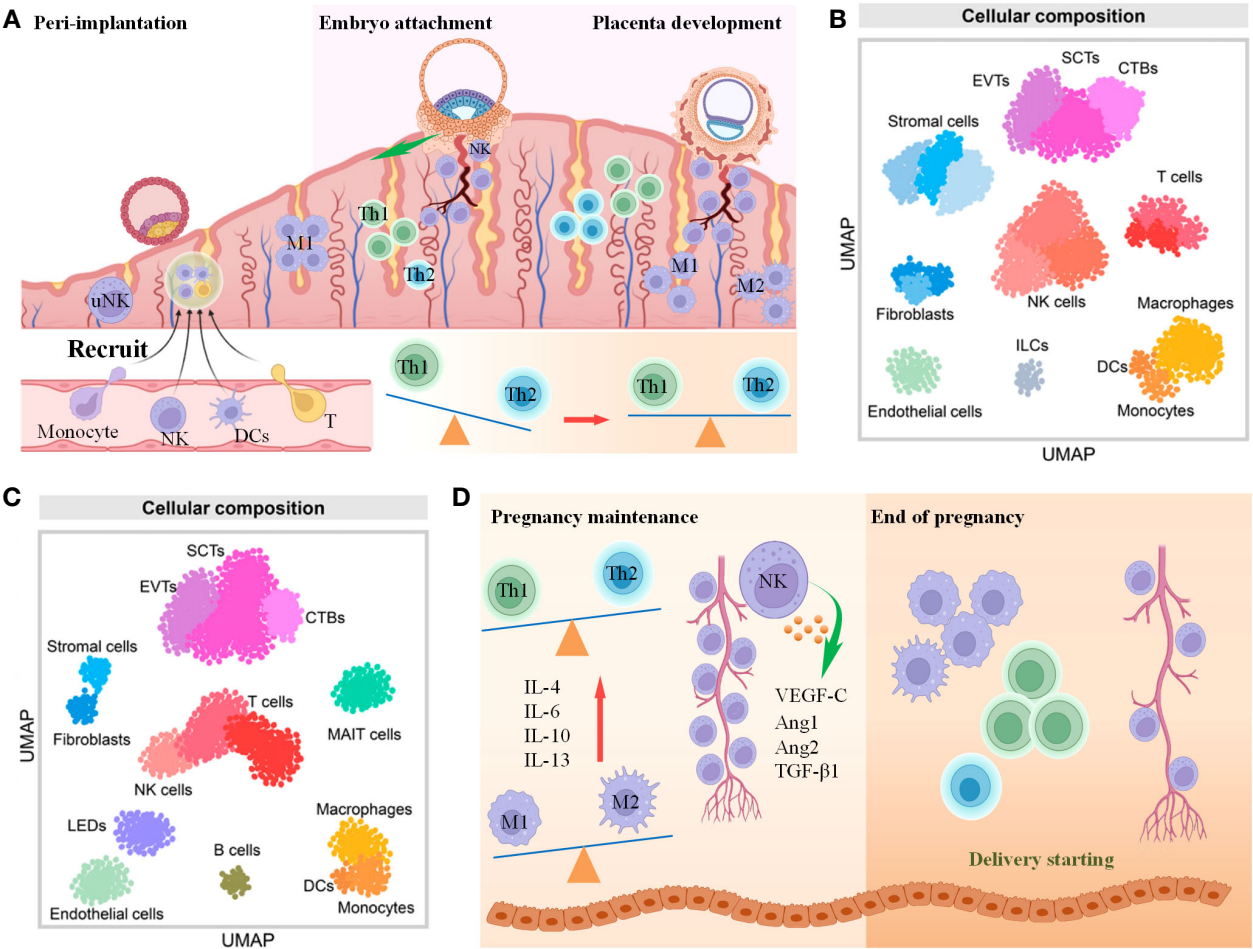
The role of immune cells in embryo implantation and development. **(A)** Scheme of immune cells changes from preimplantation to the first trimester of pregnancy **(B, C)**. UMAP showing immune cell and other cell types in the first trimester **(B)** and third trimester **(C)** of pregnancy. Cited from Derek Miller et al., 2022 (63). **(D)** Scheme of immune cells changes from the second trimester to the end of pregnancy.

Following fertilization within the fallopian tube’s ampulla, the ovum transforms into a zygote, subsequently undergoing successive rounds of cell division while traversing towards the uterus. Before embryo implantation, the endometrium needs to supply a suitable environment for immune tolerance for embryo implantation and development since the embryo is regarded as an exogenous immunogen for the maternal interface due to half of the genetic material from the father, which is also called endometrial receptivity. A large number of immune cells migrate into the endometrial tissue to regulate endometrial receptivity, accounting for 30%-40% of the total number of deciduous cells, mainly including Mφs, DCs, T cells and NKs (**Figure 4A**) (64). The dynamic changes of these immune cells play an important role in maintaining the embryo development in different stages of pregnancy, with their underlying molecular mechanisms gradually being unveiled. Furthermore, scRNA-seq technology was also used to reveal the map changes of different immune cell groups at the maternal-fetal interface during pregnancy (**Figures 4B, C**) (63), which will benefit researchers to better understand the immune cell function at the maternal-fetal interface.

### 3.1 NKs in endometrial receptivity regulation

Among them, uNKs (also called decidual NK; dNK, in decidual endometrium) are the main component of endometrial immune cells, accounting for approximately 70% of deciduous leukocytes (65). Abnormal quantities of NKs and functions are related not only to the failure of pregnancy establishment but also to placental disorders such as PE, RIF, intrauterine growth restriction and placental hyperplasia (66, 67). The luteal phase (also known as the mid-secretory phase) is a crucial stage during which the uterine endometrium prepares for pregnancy. The physical proximity of uNK cells to decidual cells, their precise upregulation in density during the implantation window, and their ability to release cytokines known to stimulate decidual development all indicate the pivotal role of uNK cells in early pregnancy events (68). During the proliferative phase of the menstrual cycle, the density of uNK cells remains relatively low and stable. However, in response to the surge of luteinizing hormone, the density of uNK cells begins to increase during the first half of the secretory phase, exhibiting a substantial 6-10-fold increase in the latter half of the secretory phase (69, 70). The dynamic changes in subpopulations of uNK cells, along with their secretion of specific cytokines such as IFN-γ, IL-10, and IL-6 at distinct time points, regulate the immune microenvironment within the endometrium (71, 72). Additionally, these cells release immunosuppressive cytokines that inhibit the activity of other immune cells, thereby maintaining immune balance. uNK cells tend to secrete pro-inflammatory cytokines resembling Th1-type cell cytokines, while simultaneously inhibiting the anti-inflammatory Th2-type cell cytokines necessary for maintaining a healthy pregnancy (73, 74). Moreover, within the endometrium, uNK cells can selectively induce cytotoxic effects by releasing cytotoxic granules like perforin and granzymes, which can selectively target and eliminate decidual cells (75). These characteristics of uNK cells play a significant role in regulating endometrial receptivity and the functions of decidual cells. A study has shown that patients with RIF have elevated CD57+/CD56+ uNK in endometrial and peripheral blood, suggesting that abnormally increased CD57 expression in uNK cells will lead to adverse pregnancy outcomes (76). Research indicates that patients with RM tend to exhibit a higher proportion of circulating activated T lymphocytes, elevated cytotoxic NK cell levels, and decreased numbers of circulating IL-10 CD56+ NK cells. Additionally, lower levels of IL-4, IL-10, and TGF-β have been observed in these individuals (77). Given the challenges in obtaining uterine endometrial samples, our understanding of uNK cell research remains limited. This constraint may restrict our comprehensive comprehension of the precise roles and functions of uNK cells in regulating the immune microenvironment of the endometrium.

**Figure 5:**
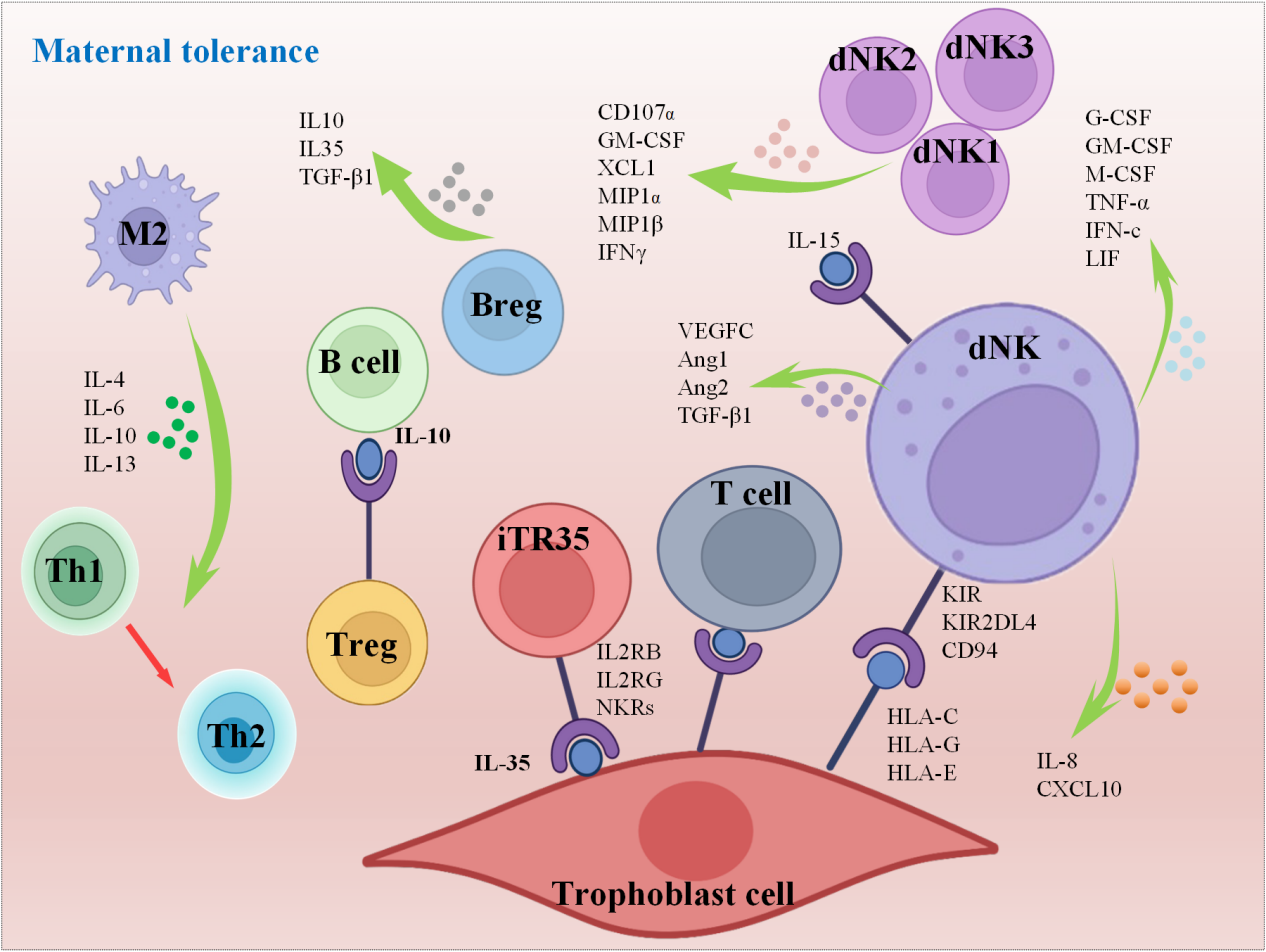
Mechanisms of maternal tolerance.

While the precise role of dNK cells remains partially understood, existing research findings indicate that cytokines secreted by dNK cells may be involved in remodeling blood vessels to create a favorable microenvironment for the fetus (**Figure 5**) (65, 78). scRNA-seq sequencing and high-throughput transcriptional sequencing were used to reveal the characteristic distribution of dNK cells in decidual tissue in patients with RSA and suggested that the polarization process of dNK cells in decidual tissue was found to be seriously disturbed in RSA compared with normal pregnant women (5). This study also demonstrated that the reduced expression of KIRs during the differentiation of dNK cells in decidual tissue may be the key reason for the high cytotoxic reactivity of polarized NK cells in RSA patients (5). Recent research discoveries have highlighted that G-CSF, GM-CSF, M-CSF, TNF-α, IFN-c and LIF are all secreted by dNK cells to regulate endometrial tolerance (79, 80). In addition, dNKs are also a major source of VEGF-C, Ang1, Ang2, and TGF-β1, which are involved in endometrial angiogenesis (81). Generally, uNKs are considered as the key population of immune cells that regulate endometrial immune tolerance to embryos before implantation, although members of the major histocompatibility complex family in human HLA-G are also involved in regulating the immune response of endometrial immune cells, influencing the production of cytokines and growth factors, inducing immunosuppression, and thus affecting endometrial receptivity (82, 83). Interestingly, the heterogeneity of NKs makes it more complex and comprehensive to evaluate the modulation of immunity on pregnancy. Decidual inherent NKs can be divided into five groups, dNK1, dNK2, dNK3, dILC2 and dNKp, through flow mass spectrometry. Among them, groups dNK1, dNK2 and dNK3 are suggested to secrete cytokines, including CD107α, GM-CSF, XCL1, MIP1, MIP 1β and IFNγ, which are responsible for interactions with other types of cells to affect endometrial receptivity, while dNKp may serve as a subtype of proliferation NKs (**Figure 5**) (84).

### 3.2 B cells in endometrial receptivity regulation

In contrast to other immune cell populations, B cells are relatively scarce within the female reproductive tract, and their abundance does not appear to vary significantly throughout the menstrual cycle (85). Nonetheless, they play a pivotal role by releasing a diverse array of immune regulatory molecules that contribute to the regulation of the IME at the maternal-fetal interface, including cytokines and chemical mediators. A particularly noteworthy regulatory molecule is the IgG4 subclass of antibodies, which exerts inhibitory effects on inflammatory reactions and the activation of immune cells. This contributes to sustaining immune tolerance and equilibrium at the maternal-fetal interface (86). Furthermore, B cells facilitate the presentation of antigens through MHC class II molecules to CD4+ T cells, leading to the suppression of T cell activity. This observation aligns with earlier research findings and underscores the multifaceted roles that B cells assume in orchestrating immune responses. Specifically, while the body of research pertaining to the immunomodulatory role of regulatory B cells (Breg cells) remains somewhat limited, recent discoveries have started to unveil their involvement in the regulation of endometrial receptivity. Breg cells are immunosuppressive cells that support immunological tolerance. Generally, Breg cells are recognized for their ability to curtail the proliferation of cytotoxic T cells and other pro-inflammatory lymphocytes by generating cytokines such as IL-10, IL-35, and TGF-β (87, 88). Notably, aside from their role in T cell suppression, these cells have been identified as one subtype of antigen-presenting cells (APCs), raising questions about their unique capacity to tolerate trophoblast antigens during the crucial stage of embryo implantation (89). Furthermore, a study observed higher CD19(+) CD24(hi) CD27(+) Breg cell frequency in peripheral blood of normal pregnancy onsets but not in spontaneous abortions by flow cytometry, indicating its partition in gestation maintenance in early pregnancy (90), and a meaningful research provide evidence that Breg cell suppression to trophoblast antigens to establish fetal-maternal tolerance in mice was mediated by glycan through CD22–LYN inhibitory signaling (89). In summary, B cells contribute to maintaining the IME of maternal-fetal immune balance through antibody production, secretion of immune regulatory molecules, interaction with immune cells, and regulation of surface molecules.

### 3.3 Other IME regulation in endometrial receptivity

As mentioned above, the dynamic fluctuations within various subsets of Mφs play a critical role in regulating T cell differentiation and immune responses, and this ongoing process continues throughout pregnancy, underscoring the significant contribution of macrophages in upholding endometrial receptivity. Simultaneously, macrophages exert control over the immune responsiveness of endometrial cells through the secretion of cytokines like IL-1β, IL-6, and TNF-α (91). Furthermore, they engage in the synthesis and release of factors such as EGF, FGF, progesterone, and bioactive lipids including prostaglandins and leukotrienes. These substances possess the capacity to alter the differentiation status of endometrial cells while also supporting the processes of endometrial vascularization and glandular development (92–95). In the decidualization of the endometrium, CD3-negative lymphocytes can interact with stromal fibroblast cells through high expression of chemotaxis, IL2RB, IL2RG and NKRs to maintain endometrial homeostasis and prepare for embryo acceptance (96). Mast cells are also essential for the regulation of endometrial tolerance. It has been shown that mice lacking mast cells develop histological changes in the endometrium that are detrimental to embryonic implantation, and these changes will further interfere with metaphase and trophoblast invasion (97). Naturally, nonimmune cells can also modulate immune cells. Researchers found that placental trophoblasts can produce the immune regulatory factor IL-35, which can induce Tconv cells to differentiate into immunosuppressive iTR35 cells by activating STAT1 and STAT3 (**Figure 5**). It is essential for the establishment of an immune tolerance microenvironment at the maternal-fetal interface and the maintenance of pregnancy (98). Additionally, DCs can also interact closely with other immune components, such as NKs and Mφs, as well as the endocrine system to maintain a pregnancy-friendly environment, while abnormal DCs can also lead to APOs (99).

In general, immune cells play a crucial role in regulating the receptivity of the uterine lining through close interactions and molecular signaling, ensuring the successful implantation and development of the embryo. However, the specific mechanisms of action of immune cells and their regulatory modes in different physiological and pathological contexts require further investigation. Gaining a deeper understanding of how immune cells modulate the receptivity of the uterine lining will contribute to the development of more effective intervention strategies, enhancing female fertility. This understanding also opens up new avenues and approaches for addressing infertility and other reproductive health issues.

## 4 Dynamic alterations of the immune profile in embryo implantation and development

Once the zygote undergoes development and transforms into a blastocyst, it begins to directly interact with the maternal interface physically, forming the maternal-fetal interface, which is also called the process of embryo implantation. The maternal-fetal interface is the part of the endometrium and extraembryonic tissue formed in the complex process of blastocyst implantation and is considered the direct communication interface between the mother and fetus, which is mainly composed of trophoblast cells, decidual cells and immune cells (100). In essence, the maternal-fetal interface stands as a remarkable testament to the exceptional synergy among trophoblast cells, decidual cells, and immune cells. Immune cells, including NKs, Mφs, and T cells, are an indispensable component of this intricate ecosystem. The intricate molecular dialogues between these immune cells and decidual and trophoblast cells delicately balance tolerance and protection, and this precise coordination is of utmost importance for shielding the developing embryo from rejection while simultaneously facilitating its optimal growth and development.

### 4.1 NKs in embryo implantation and development

During embryo implantation, moderate migration and invasion of the blastocyst into the endometrium and spiral artery remodeling are widely believed to be the critical steps for successful pregnancy and are regulated by immunomodulation to a large extent. Any factors causing the disorder of IME at this period may lead to APOs, including RSA, infection, PE and premature birth, and intrauterine growth restriction (63, 101, 102). Researchers observed the failure of spiral arterial remodeling in uNK knockout mice, there is an observed failure in spiral arterial remodeling, which also results in a limitation of uNK expansion, indicating its essential role in embryo development (103, 104). Notably, Anna Sliz et al. creatively identified the Gab family members Gab3 as a key determinant of NK cell expansion, while loss of Gab3 resulted in impaired IL-2 and IL-15–induced NK cell priming and expansion due to a selective impairment in MAPK signaling (104). For the first week of pregnancy, uNK cells infiltrate and aggregate around the spiral artery (**Figure 4A**), but their total amount does not change significantly as the embryo develops. Until the late stage of pregnancy, the number of uNKs was significantly reduced, indicating that the enrichment of uNKs coincided with the invasion period of trophoblast cells (105). This is because the receptors CD94/NKG2A, KIR2DL4 and KIR expressed by uNK cells specifically bind to HLA-E, HLA-G and HLA-C on trophoblasts, respectively, to promote their invasion of the endometrium (**Figure 5**) (106). Moreover, the continuous differentiation of uNK is responsible for endometrial regeneration and successful pregnancy (107).

The release of uterine IL-15 promotes dNKs maturation and mature dNK cells are an important source of growth factors and cytokines, which can regulate the invasion of trophoblast cells and reshape the function of spiral arteries through a variety of cytokines (108, 109). For example, dNKs can secrete VEGFC to initiate lymphatic mimicry in maternal endothelial cells to benefit spiral artery remodeling in mice by stimulating VEGFE3 pathway signal transduction (110) as well as IFNγ, VEGF, and TNFα to promote decidual remodeling and blastocyst implantation (111), the release of IL-8 and CXCL10 by dNK can also promote extravillous trophoblast invasion (4). Above all, it is obvious that dNKs have multiple regulatory functions in spiral artery remodeling, differentiation and invasion of trophoblast cells, fetal development and maternal immune tolerance in early pregnancy. Extremely interesting research verified the existence of memory in uNKs, which means more significant placental growth-promoting activity and the ability to maintain pregnancy while receiving stimulation again (112). Importantly, the process of embryo implantation and development is accompanied by the precise balance regulation of immune tolerance at the maternal-fetal interface, that is, to control the balance of pro-inflammatory and anti-inflammatory at the local microenvironment.

Preimplantation and implantation mainly promote local inflammatory reactions to mediate cell proliferation, induce trophoblast invasion, promote decidualization of endometrial stromal cells and form a large number of tiny vessels, while during the first and second trimesters of pregnancy, chemokines inducing the increase in anti-inflammatory factors to induce immune tolerance and maintain pregnancy are largely needed, and inflammatory cytokines are activated again at the mother-fetal interface at the late stage of pregnancy, promoting the activation of the delivery mechanism (113, 114).

### 4.2 Mφs in embryo implantation and development

**Figure 6:**
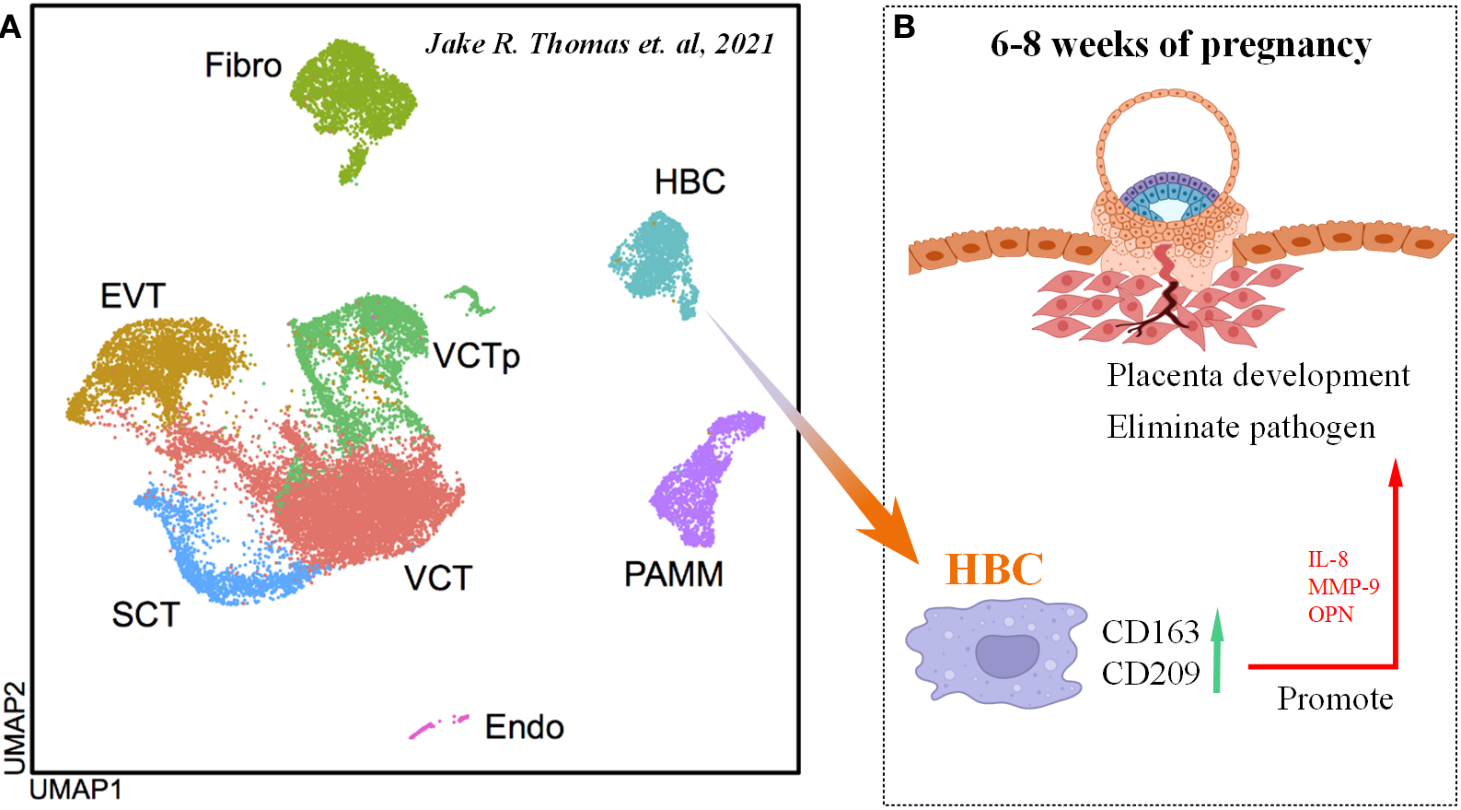
Origin and function of HBC cells. **(A)** UMAP of various cell types in the embryo scRNA-seq data. Cited from Jake R. Thomas et al., 2021 (115). **(B)** Scheme of HBC functions in maternal-fetal interface.

Interestingly, researchers identified a group of fetal-derived Mφs by single-cell RNA sequencing (named Hofbauer cells, HBC) in the placenta at 6-12 week after fertilization (**Figure 6A**) (115), which are M2-like Mφs with high expression of CD163 and CD209 (116). Based on these findings, researchers identified another group of maternally derived Mφs, named PAMMs, which can be divided into three subgroups: FOLR2-CD9hiCCR2lo/int (PAMM1a), FOLR2-CD9-/intCCR2+ (PAMM1b) and HLA-DRhi/FOLR2hi (PAMM2). In detail, placental surface-specific resident PAMM1a is believed to release MMP-9 and fibronectin to participate in damaged tissue repair, monocytes such as PAMM1b have the ability to secrete IL-1β to induce an inflammatory response, and PAMM2s are decidual Mφs (DMs) (115). Cytokines secreted by HBC (such as IL-8, MMP-9, OPN) can promote the growth and development of the placenta (**Figure 6B**), maintain homeostasis, and participate in eliminating pathogenic microorganisms and bacteriophages *in vitro*; in contrast, their abnormal function can cause chorioamnionitis, spontaneous abortion and metabolic diseases in the fetus (115, 117).

In the early stage of embryo implantation, various chemokines and cytokines are secreted at the maternal-fetal interface, and peripheral blood mononuclear cells are recruited to differentiate into DMs; then, DMs self-renew and proliferate during embryo development (118). As shown in **Figures 4A–D**, DMs exhibit different phenotypes and functions in the decidual microenvironment at different stages of pregnancy, and this dynamic change plays an important role in the establishment and maintenance of normal pregnancy (119). In detail, during the implantation period of the embryo, the uterus presents a local inflammatory state, and GM⁃CSF induces the polarization of DMs to the M1 type, forming an inflammatory environment in the uterus to promote the adhesion of the embryo to the endometrium. Throughout the first to second trimesters of pregnancy, DMs gradually convert to the M1/M2 mixed phenotype as trophoblasts invade the endometrial stroma, and DMs secrete VEGF to participate in angiogenesis and spiral artery remodeling in preparation for fetal blood supply. After placental development is completed, DMs are polarized from the M1/M2 mixed to the M2 type to maintain an anti-inflammatory environment *in utero* and prevent fetal rejection by the mother, as well as engulf trophoblast cell fragments and apoptotic cells (**Figure 4D**) (120). Before delivery, the endometrium aggregates M1-type Mφs, which secrete proinflammatory factors and participate in the processes of uterine contractions, childbirth and uterine repair (**Figure 4D**) (121).

Naturally, abnormal activation of Mφs leads to changes in the IME of the maternal-fetal interface, which interferes with normal development processes, such as placental vascular development, trophoblast cell invasion and spiral artery remodeling, and leads to adverse pregnancy outcomes, including RIF (122), RSA (123), fetal growth restriction (124), premature delivery and PE (117). Therefore, how to effectively regulate the polarization of Mφs at the maternal-fetal interface and prevent and treat pregnancy complications is a key research direction in the future.

### 4.3 T cells in embryo implantation and development

CD4+ T cells are also recruited to the maternal-fetal interface under the action of chemokines during implantation and early pregnancy, which mediates the establishment and maintenance of the immune tolerance balance of the maternal-fetal interface (125). CD4+ T cells in the maternal-fetal interface are mainly divided into Th cells and Tregs. Toward different immune threats, Th cells can make different responses and differentiate into Th 1, Th 2, Th 17 or Th 22 cells (126).

Similar to the polarization of Mφs, the balance of Th 1/Th 2 is the key to maintaining the homeostasis of the maternal-fetal interface, which can be regulated by various molecules and manifested in different states at different stages of pregnancy (**Figures 4A–D**). Studies have shown that the imbalance of Th 1/Th 2 can lead to RIF, PE, and even RSA, and the fundamental reason for this consequence is the disorder of the microenvironment of the maternal-fetal interface (127–131). During the preimplantation and implantation periods, Th1 cells are the main population to function (**Figure 4A**) and secrete proinflammatory factors, including IFN-γ, TNF-α and IL-2, which promote moderate local inflammation and induce trophoblast cells to invade the endometrium. In contrast, in the early to middle periods of pregnancy, Th2 cells play a dominant role by secreting IL-4, IL-6, IL-10, and IL-13 to inhibit local inflammation and protect the fetus from maternal rejection (132). As the embryo develops, Th1 once again dominates before delivery and starts the delivery process (**Figure 4D**) (133).

In addition, the balance between Th17 and Treg cells can also regulate the implantation and development of embryos. Maternal Treg cells mainly express CD4+, CD25+ and Fox3+ to protect against Th17-mediated immune responses, thereby suppressing their own immune response to paternal antigens and contributing to the maintenance of pregnancy (134, 135). Th17 cells can specifically express RORγ and secrete cytokines such as IL-17, IL-21 and IL-23, which play an important role in promoting inflammatory reactions and participate in autoimmune diseases (136). During normal pregnancy, the balance of Treg/Th17 tends toward Treg cells. Once more Th cells in the endometrium differentiate into the Th17 type, the Treg/Th17 imbalance and endometrial immune microenvironment will be disordered, which eventually leads to APOs (137–139). As a result, CD4+ T cells play an important role in the establishment and maintenance of immune tolerance balance at the maternal-fetal interface. Except CD4+ T cells, CD8+ T cells involved in the regulation of process during embryo implantation and development. As mentioned earlier, CD8+ T cells were commonly suppressed to maintain immune tolerance which benefiting embryo implantation during early pregnancy stage. However, the infiltration of CD8+ T cells in decidua function in the stages of embryo implantation and development. Paula et al. observed that decidual CD8+ T lymphocyte supernatants increased the capacity of extravillous trophoblast cells to invade through Matrigel which promoting the implantation of embryo (140). In normally progressing pregnancies, Blois et al. found that adequate levels of progesterone led to a predominance of pregnancy protective cytokines, possibly via PIBF secreted by uterine CD8+ T cells, while depletion of CD8+ T cells resulted abortions, which indicating the essential roles of CD8+ T cells in pregnancy maintenance (141).

### 4.4 HLA molecules in embryo implantation and development

Despite immune populations, trophoblasts can also express embryo antigens, secrete cytokines, and form immune tolerance to escape the attack of the maternal immune system, while the imbalance of this escape mechanism may lead to implantation failure of the embryo. Researchers found that members of the family of major histocompatibility (HLA) complexes in humans, including 3 soluble isoforms (sHLA-G5, G6, G7) and 4 membrane-bound isoforms (HLA-G1, G2, G3, G4), were highly selectively expressed by trophoblast cells outside the maternal-fetal interface and can dynamically regulate the process of embryo implantation and development through changes in their expression levels (142, 143). Studies have shown that the levels of sHLA-G in women’s blood are positively related to the success of *in vitro* fertilization. In contrast, low expression of HLA-G indicates a high risk of RSA (144–148).

**Figure 7:**
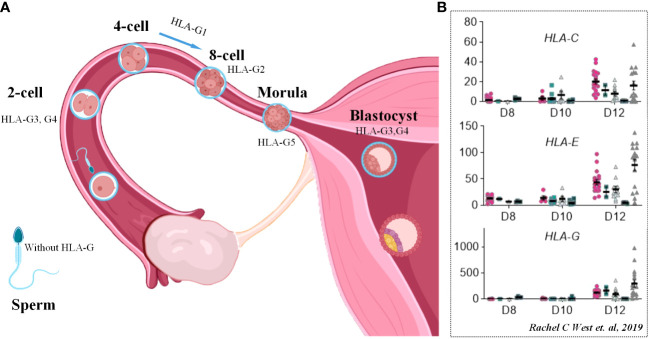
HLA molecules in embryo implantation and development. **(A)** Scheme of dynamic process of HLA-G molecule expression in pre-implantation stage of human embryo. **(B)** FPKM values of MHC class I genes (HLA-C, HLA-E and HLA-G) in human embryos between D8 and D12. Cited from Rachel C West et al., 2019 (149).

Among the members of HLA-G, HLA-G3 and HLA-G4 are the main transcripts throughout the preimplantation stage, followed by HLA-G1, HLA-G2, and HLA-G5, while HLA-G6 does not appear until the blastocyst stage. After implantation, HLA-G1 and -G5 expression differentiates toward the trophoblast ectoderm and gradually disappears in the inner cell mass during development (**Figure 7A**) (150). The physiological function of HLA-G has also been revealed. HLA-G promotes the production of beta human chorionic gonadotrophin by activating the Erk 1/2 pathway, which has a positive impact on embryo implantation (151). Isoform HLA-G 5 promotes the invasion of primary trophoblast cells, the trophoblast cell line JEG-3 and JAR cells by activating phosphorylation of the ERK pathway (144). *In vitro* studies have confirmed that HLA-G can bind to specific receptors expressed by uNKs and plays an important role in the invasion behavior of the endometrium by trophoblasts (152).

In addition, trophoblasts also express classical HLA class I molecules HLA-C and nonclassical HLA class I molecules such as HLA-E and HLA-F, which are of great significance to the immune microenvironment of the maternal-fetal interface (153). Evidence from single-cell RNA sequencing clarified the upregulation of HLA-C, HLA-E and HLA-G on the 12th day of migrating trophoblasts, which is considered the process of embryo implantation (**Figure 7B**) (149).

### 4.5 Other immune cells in embryo implantation and development

Although other immune cells are present in smaller numbers in the endometrium, their regulation of the maternal-fetal interface during pregnancy by participating in embryonic immune protection, regulation, and development through multiple mechanisms still plays a crucial role in embryo implantation and development.

Uterine DCs (uDCs) recognize antigens through their surface antigen recognition receptors and, after capturing them, process and degrade the antigens into fragments. These antigen fragments are then presented to T cells through major histocompatibility complex (MHC) molecules (MHC-I molecules present endogenous antigens to CD8+ T cells, while MHC-II molecules present exogenous antigens to CD4+ T cells) (154, 155). Simultaneously, uDCs express co-stimulatory molecules such as CD80 and CD86, which interact with relevant receptors on the surface of T cells (such as CD28), providing a second signal that promotes T cell activation and proliferation (156). uDCs can also express immune regulatory molecules such as PD-L1. After PD-L1 on uDCs binds to the PD-1 receptor on the surface of T cells, it transmits inhibitory signals that suppress T cell activation and immune responses (157). It also inhibits inflammatory reactions and Treg cell immune responses by secreting various immune regulatory cytokines, such as IL-10 and TGF-β (158). Additionally, B cells can interact with Treg cells, promoting the expansion and activation of Treg cells, which in turn suppresses the immune system’s attack on the fetus (159). And the expression of certain surface molecules on B cells at the maternal-fetal interface, such as CD69 and PD-L1, can be regulated to activate or inhibit other immune cells (155). During embryo development, B cells also can differentiate into plasma cells and produce hematopoietic cells such as red blood cells, white blood cells, and platelets, which supports the development and function of the embryo hematopoietic system, especially in the early stages of embryo development (160, 161). In addition to regulating embryonic development through binding to HLA-C ligands on the surface of embryonic cells, NKs also secrete IFN-γ, which can regulate the types and activity of immune cells in the placenta and endometrium, which affects the process of embryo implantation and development (162). NKs can promote the proliferation and migration of endothelial cells and the formation of blood vessel lumens by secreting cytokines such as vascular endothelial growth factor and matrix metalloproteinases. Additionally, they selectively eliminate abnormal spiral artery cells, thereby promoting the remodeling of spiral arteries (163, 164). In addition, NKs possess the ability to identify potential abnormal embryos by binding to specific ligands in the embryo, controlling selective immune clearance of the embryo.

## 5 Conclusion and perspectives

By presenting the status of the IME at each stage of reproductive process, combined with the application of scRNA-seq technology in these stages, we have systematically summarized the important role of dynamic balance of IME in promoting successful pregnancy and the serious adverse consequences caused by immune regulation disorder. However, the advantage of scRNA-seq lies in its capacity to unveil intricate tissue composition details and dynamic changes, while it falls short of revealing the functional roles and molecular mechanisms of specific identified cell types. Moreover, the information yielded by scRNA-seq is voluminous, and the analytical strategies employed hold a pivotal influence over result interpretation. Hence, it is imperative to approach scRNA-seq outcomes with objectivity, anchored in empirical validation. Immune cells resident in the uterus or ovary and recruited from peripheral blood are involved in almost every stage of pregnancy, especially after fertilization, by direct or indirect interactions with other parenchymal cells and stromal cells to exclude signal transduction to ensure the coordination of all components of the complex regulatory networks so that the normal reproductive process can be carried out. Overall, we summarize the critical factors influencing female fertility to highlight the importance of the IME constructed by both immune populations and nonimmune cells through cytokines in female reproductive process. The immune system plays an indispensable role in the female reproductive process, encompassing ovarian follicle formation, ovulation, embryo implantation in the uterine lining, and embryonic development. Immune cells engage in intricate molecular communication networks to regulate each critical stage. Mφs, NKs, B cells, T cells, and other immune components collaborate synergistically, not only influencing the development of reproductive cells but also ensuring the receptivity of the uterine lining, successful embryo implantation, and the smooth progression of pregnancy. However, despite significant progress, there remain many unknowns regarding the precise roles of these immune cells, their molecular mechanisms, and their interactions in diverse physiological and pathological contexts. Future research should delve deeper into these matters, particularly focusing on interactions and regulatory pathways among different immune cell subtypes. In addition, more attention should be given to research on the treatment of various APOs or infertility through immunotherapy to improve the immune abnormality and to further improve female fertility, and more technologies with higher throughput should be utilized to acquire more information about not only the expression change of some signal molecules but also the alterations of time and space point at one disease model or one physiological process at the same time so that we can obtain more comprehensive knowledge about the female reproductive process, which will benefit the development of treatment methods in the clinic.

## Author contributions

MD and YX: conceptualization, investigation, data curation, writing –original draft, visualization, GG: writing – review & editing. YZ: conceptualization, writing – review & editing, funding acquisition. All authors contributed to the article and approved the submitted version.
